# Comparison of artificial intelligence-adjudicated spatiotemporal dispersion in spontaneous vs. induced atrial fibrillation among patients with persistent AF

**DOI:** 10.1007/s10840-025-02122-8

**Published:** 2025-09-08

**Authors:** Jonathan Alfred, Giulio Mastella, Isabel Rattka, Niklas Jurik, Eimo Martens, Karl-Ludwig Laugwitz, Manuel Rattka

**Affiliations:** 1 School of Medicine and Health, Department of Clinical Medicine—Clinical Department for Cardiology, University Medical Centre, Technical University of Munich, Munich, Germany; 2 European Reference Network Guard Heart, European Union, Munich, Germany; 3 German Centre for Cardiovascular Research (DZHK), Partner Site, Munich, Germany

## Introduction

Pulmonary vein isolation (PVI) is the standard ablation strategy for paroxysmal atrial fibrillation (AF), but the best approach for persistent AF (persAF) remains uncertain. It recently has been shown that combining PVI with ablation targeting spatiotemporal electrogram dispersion improves outcomes over PVI alone in persAF patients [1]. However, it is unclear whether this strategy is applicable to persAF patients presenting in sinus rhythm after cardioversion, where AF induction would be required prior to ablation. This analysis aimed to determine whether spatiotemporal dispersion patterns differ between spontaneous AF (sAF) and induced AF (iAF).

## Methods

In patients with ongoing persAF scheduled for PVI plus dispersion-targeted ablation, first, a high-density electroanatomic (EA) map of the left atrium (LA) was created during spontaneous AF (sAF). Next, electrical cardioversion was conducted and a second EA map was generated during paced rhythm. In patients in whom pacing re-induced AF (iAF), another high-density map was acquired, starting approximately 5 min after AF induction. Spatiotemporal dispersion was identified and tagged using the Volta AF-Xplorer system (VX1, Volta Medical, Marseille, France) in sAF and iAF maps.

The LA was divided into 16 standardized subregions for analysis as previously described by Seitz et al. [2]. Two analyses were conducted: A binary comparison assessed the presence or absence of dispersion in each subregion between sAF and iAF. A semi-quantitative score evaluated dispersion extent per region: 0, none; 1, sparse(< 10% of surface); 2, moderate(10–50%); 3, extensive(> 50%) [2]. The McNemar test assessed binary differences; Wilcoxon test was used for ordinal data. Ablation included PVI and dispersion-targeted ablation based on the iAF map. Follow-up included 7-day Holter-monitoring at 3, 6, and 12 months. The study was approved by the local ethics committee.

## Results

Ten patients (median age 75 [64–82] years; 40% female; 50% with long-standing persAF) underwent both sAF and iAF mapping. Each map was divided into 16 subregions, totaling 160 subregions per mapping condition for analysis.

**Fig. 1:**
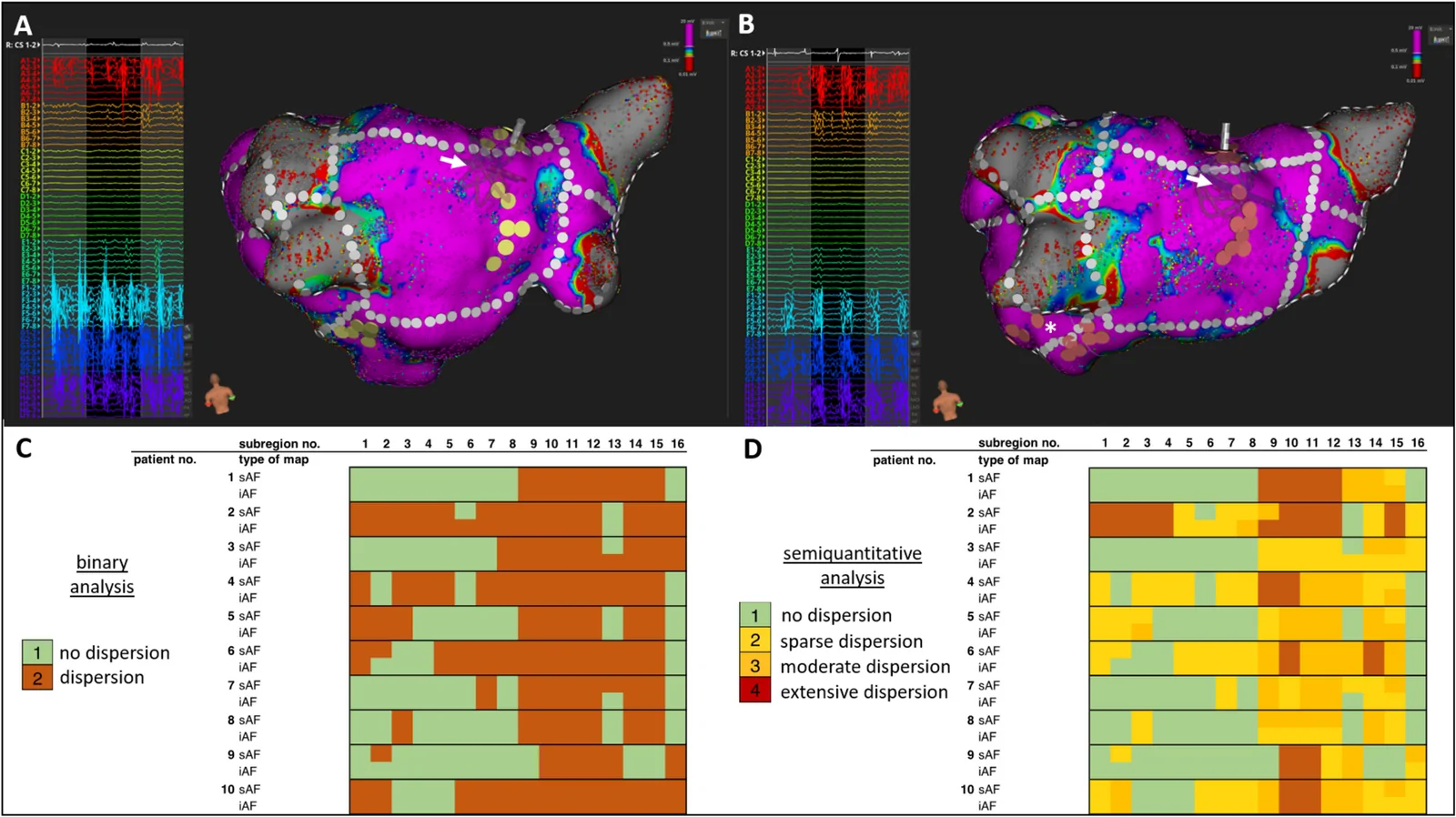
**A** Posterior view of the segmented left-atrial EA map in sAF for patient three. Grey dots divide the subregions; yellow dots mark areas of dispersion. The shadow of the high-density mapping catheter (white arrow) is visible near the roof. Representative intracardiac electrograms demonstrate dispersion. **B** Posterior view of the same region mapped in iAF. Red dots indicate dispersive areas, and intracardiac electrograms again show dispersion with the catheter in a similar position (white arrow). While, for example, regions 10 (roof), 11 (posterior wall), and 12 (left atrial floor) are classified identically, region 13 (mitral isthmus) shows dispersion in iAF (white asterisk) but not in sAF. **C** Binary comparison of each sub-region per patient, depicting the presence (1 = red) or absence (0 = green) of spatiotemporal dispersion. **D** Semiquantitative dispersion score per subregion: 0 = no dispersion (green); 1 = sparse dispersion, < 10% of tags (yellow); 2 = moderate dispersion, 10–50% of the sub-region’s surface (orange); 3 = extensive dispersion, > 50% (red)

In the binary analysis (Fig. 1C), spatiotemporal dispersion was detected in 98 of 160 sAF subregions and in 97 of 160 iAF subregions. In 95 subregions with dispersion, the classification was identical in both sAF and iAF. Only 5 out of 160 matching subregions showed a discrepancy in classification between sAF and iAF.

In five patients, there was no deviation at all in the classification of matching subregions, while in the other five patients, a difference was observed in only one of the 16 subregions, respectively (e.g., patient number 3; Fig. 1A and B).

In the semi-quantitative analysis (Fig. 1D), 140 of 160 matching subregions (87.5%) retained a consistent classification between sAF and iAF. A one-category discrepancy occurred in 20 subregions (12.5%), with no instance of a difference of two or more categories. In all patients, there was at least one deviation of matching subregions. In four patients, there was a one-region deviation, while there was a difference between sAF and iAF maps in two subregions in three patients, in three subregions in two patients, and in four subregions in one patient. No statistically significant difference was observed (binary *p* = 1.000; semi-quantitative *p* = 0.371).

PVI plus dispersion ablation during iAF terminated AF in five of ten patients. Three converted to sinus rhythm. Two developed macro-reentrant atrial tachycardias: one had perimitral flutter treated with a linear lesion to the mitral annulus; the other had a scar-related flutter treated with a line from the right superior pulmonary vein to the mitral annulus. In the five patients who remained in AF post-ablation, the median AF cycle length in the left atrial appendage increased from 147 [138, 164] to 175 [162, 184] ms (*p* = 0.043). No complications occurred. During the 12-month follow-up period, nine of the ten patients (90%) remained free of any atrial tachycardia recurrence.

## Discussion

In this study, we investigated whether spatiotemporal dispersion patterns differ between sAF and iAF in patients with persAF. Previous studies comparing spontaneous and induced or re-induced AF within the same patients have shown that, while the average AF cycle length generally remains similar, the activation patterns may differ [3, 4]. In contrast, our results from both binary and semiquantitative comparisons demonstrated a high degree of concordance between sAF and iAF maps, with only minor regional differences. These findings support the notion that iAF may serve as a reliable surrogate for spatiotemporal dispersion-guided substrate analysis.

Ablation targeting iAF-identified dispersion areas achieved acute procedural success in half of the patients. This is slightly lower than the 66% AF termination rate reported previously, potentially due to our left atrium-only approach [1]. In patients who remained in AF, dispersion ablation still prolonged AF cycle length, indicating modification of arrhythmogenic substrate potentially participating in the arrhythmogenic process [5]. Despite the small sample size and lack of a control group, the 12-month arrhythmia-free survival of 90% is promising, particularly in this difficult-to-treat population.

While our findings suggest that iAF can serve as a surrogate for sAF in identifying spatiotemporal dispersion via electrogram analysis tools like VX1, the mechanistic meaning of dispersion remains uncertain. Dispersion is thought to reflect conduction abnormalities such as slowing, anisotropy, or micro-reentry. However, its physiological basis is not fully understood and it remains unclear whether dispersion marks critical AF drivers or simply correlates with non-specific conduction heterogeneity [1, 2, 6].

Another important factor to keep in mind is that electrical activation patterns during iAF may vary depending on how long AF has been sustained. Studies have shown that immediately after AF induction, conduction is often more organized and becomes progressively disorganized over time [7]. Early-phase iAF may therefore not fully reflect the remodeling seen in long-standing AF. These limitations highlight the need for further studies to clarify whether dispersion is a reliable ablation target and how best to apply it in practice.

## Conclusion

In conclusion, spatiotemporal dispersion distribution and extent during iAF closely mirror those observed during sAF in patients with persAF. These findings support the use of iAF mapping as a surrogate for dispersion assessment when sAF is not present at the time of ablation.

## References

[1] Deisenhofer I, Albenque J-P, Busch S, et al. Artificial intelligence for individualized treatment of persistent atrial fibrillation: a randomized controlled trial. Nat Med. 2025;31:1286–93. 10.1038/s41591-025-03517-w PMC12003177 39953289

[2] Seitz J, Mohr Durdez T, Lotteau S, et al. Artificial intelligence–adjudicated spatiotemporal dispersion: a patient-unique fingerprint of persistent atrial fibrillation. Heart Rhythm. 2024;21:540–52. 10.1016/j.hrthm.2024.01.007 38215808

[3] Boriani G, Bartolini P, Biffi M, et al. Atrial signal analysis and defibrillation threshold assessment in chronic persistent and reinduced atrial fibrillation. J Cardiovasc Electrophysiol. 2002;13:449–54. 10.1046/j.1540-8167.2002.00449.x 12030526

[4] Saksena S, Prakash A, Krol R, et al. Regional endocardial mapping of spontaneous and induced atrial fibrillation in patients with heart disease and refractory atrial fibrillation. Am J Cardiol. 1999;84:880–9. 10.1016/s0002-9149(99)00459-2 10532504

[5] Haïssaguerre M, Sanders P, Hocini M, et al. Changes in atrial fibrillation cycle length and inducibility during catheter ablation and their relation to outcome. Circulation. 2004;109:3007–13. 10.1161/01.CIR.0000130645.95357.97 15184286

[6] Loring Z. Piccini J TAILORing AI-guided trestment for atrial fibrillation. Nat Med. 2025;31:1073–4. 10.1038/s41591-025-03611-z 40175739

[7] Ravelli F, Masè M, Del Greco M, et al. Deterioration of orrganization in the first minutes of atrial fibrillation: a beat-to-beat analysis of cycle length and wave similarity. J Cardiovasc Electrophysiol. 2007;18:60–5. 10.1111/j.1540-8167.2006.00620.x 17229301

